# The Impact of Constraint-Induced Movement Therapy on Motor Recovery in Middle Cerebral Artery Infarction Having Cardiomegaly in an Intensive Care Unit

**DOI:** 10.7759/cureus.54384

**Published:** 2024-02-17

**Authors:** Shweta A Nainani, Raghumahanti Raghuveer, Harsh R Nathani, Arasha F Khan

**Affiliations:** 1 Neuro Physiotherapy, Ravi Nair Physiotherapy College, Datta Meghe Institute of Higher Education & Research, Wardha, IND

**Keywords:** intensive care unit, gait, constraint induced movement therapy, middle cerebral artery infarct, stroke

## Abstract

Stroke can be characterized by rapidly emerging neurological manifestations of global or focal impairment of neurological functionality, with consequences lasting a day or more or giving rise to mortality, with no significant etiology other than vascular origin. A middle cerebral artery (MCA) infarct is a form of stroke that develops when the MCA, one of the primary arteries providing blood to the brain, becomes blocked or obstructed. Constraint-induced movement therapy (CIMT) is an emerging method that has mainly been utilized to rehabilitate stroke patients, especially upper extremities. According to recent advances, CIMT can also be applied to the lower limbs to increase insufficient limb balance, thereby facilitating gait. This case report is based on a 65-year-old female who had weakness in the left side of the body and slurring of speech and was diagnosed with an MCA infarct. She was managed with CIMT in the ICU along with conventional physiotherapy. The outcomes showed that CIMT is a beneficial approach for patients with stroke.

## Introduction

Strokes are the world’s second-ranked significant factor for death and the third-ranked significant form of disability [1]. A stroke is described by the WHO as a suddenly evolving cerebral presentation of a worldwide or local impairment of function in the brain, with repercussions lasting longer than 24 hours or leading to mortality, with no significant etiology beyond its vascular origin [2]. Strokes are divided into two types: ischemic (80-87%) and hemorrhagic (13-20%). Ischemic strokes are the result of thrombus, embolism, or widespread hypoperfusion [3]. Cerebrovascular accident is an epidemic issue that is the leading parameter of disability in people [4]. According to the most recent data, 16.9 million individuals experience a cerebrovascular accident each year, with a worldwide incidence of 258 per 100,000 people per year, considerable discrepancies between high- and low-income countries, and an age-adjusted epidemiology 1.5 times higher in men than in women [5]. The epidemiology of cerebrovascular accidents in low- and middle-income nations has more than quadrupled during the last four decades [6].

Ischemic strokes are categorized into three types: anterior cerebral artery, middle cerebral artery (MCA), and posterior cerebral artery. MCA infarcts are the most common and impact motor activity due to their involvement in the corticospinal tract [7]. When blood flow from the MCA, one of the major arteries in the brain, is unexpectedly halted (ischemia) or completely stopped (infarction), a stroke ensues [8]. MCA infarctions can induce symptoms such as rapid-onset paralysis or numbness on one side of the body; typically, the face, arm, or leg are impacted [9]. These symptoms can also cause linguistic difficulties, including difficulty speaking. Patients may also experience visual problems, such as hemianopia. Cognitive skills may be affected, resulting in disorientation or confusion [10]. Aging, ethnicity, racial or ethnic background, and ancestry are all irreversible predisposing parameters for stroke [11]. Among other infectious agents, bacterial pathogens are the most prevalent cause of stroke [12]. Flexible stroke risk factors include elevated blood pressure, atrial fibrillation, various cardiac problems, hyperlipidemia, diabetes, smoking, lack of exercise, carotid stenosis, and transient ischemic attack [13].

Constraint-induced movement therapy (CIMT) was invented to address upper extremity limitations following a cerebrovascular accident and is a widely researched strategy for patient physiotherapy. However, recent advances suggest that CIMT also applies to the lower limbs for balance and gait. The original CIMT comprises non-paralyzed arm constraints and task-specific training. Modified types also use non-paralyzed arm restricting, although not as intensely as the primary CIMT. Both original and adapted CIMT positively affected motor activity, arm-hand functions, and self-experienced arm-hand activities in daily life [14]. It is anticipated that individuals who can benefit significantly from CIMT account for at least 50% of the overall stroke population [15]. Although meant to improve upper arm function, numerous authors have found that it improves balance [16]. CIMT, a stroke rehabilitation strategy, necessitates rigorous training of the damaged arm while restricting the mobility of the unaffected arm for six hours daily, five days a week, for two weeks [17]. The technique uses behavioral psychology, motor learning, and skill development concepts [18].

## Case presentation

Patient information

The patient was a 65-year-old female who was brought to the neuro-outpatient department for complaints of weakness in the left side of the body and slurring of speech. She experienced these complaints for one day. She reported similar complaints six months ago and thereby visited the local hospital, where she was treated with medications. After a month, she experienced weakness on the left side of her body and was again treated with medications. She was admitted to the neurology ward, where specific investigations were done, such as an ECG, a two-dimensional echocardiogram, a color Doppler, an MRI, a complete blood count, a CT scan, and a chest X-ray. The patient had a known case of rheumatic heart disease, mild mitral stenosis, and mitral regurgitation; had a history of bronchial asthma and hypertension for three years; and was on medication for the same. The patient had no history of giddiness or falls. Moreover, there is no record of a head injury, fever, seizures, loss of consciousness, or headache in her medical history. She was then shifted to the medicine intensive care unit (MICU) for four days, then moved to the ward and treated with medications given, including a tablet of Clopitab-A 150 mg, a tablet of Rosuvas 40 mg, and an injection of Optineuron intravenous.

Clinical findings

**Figure 1:**
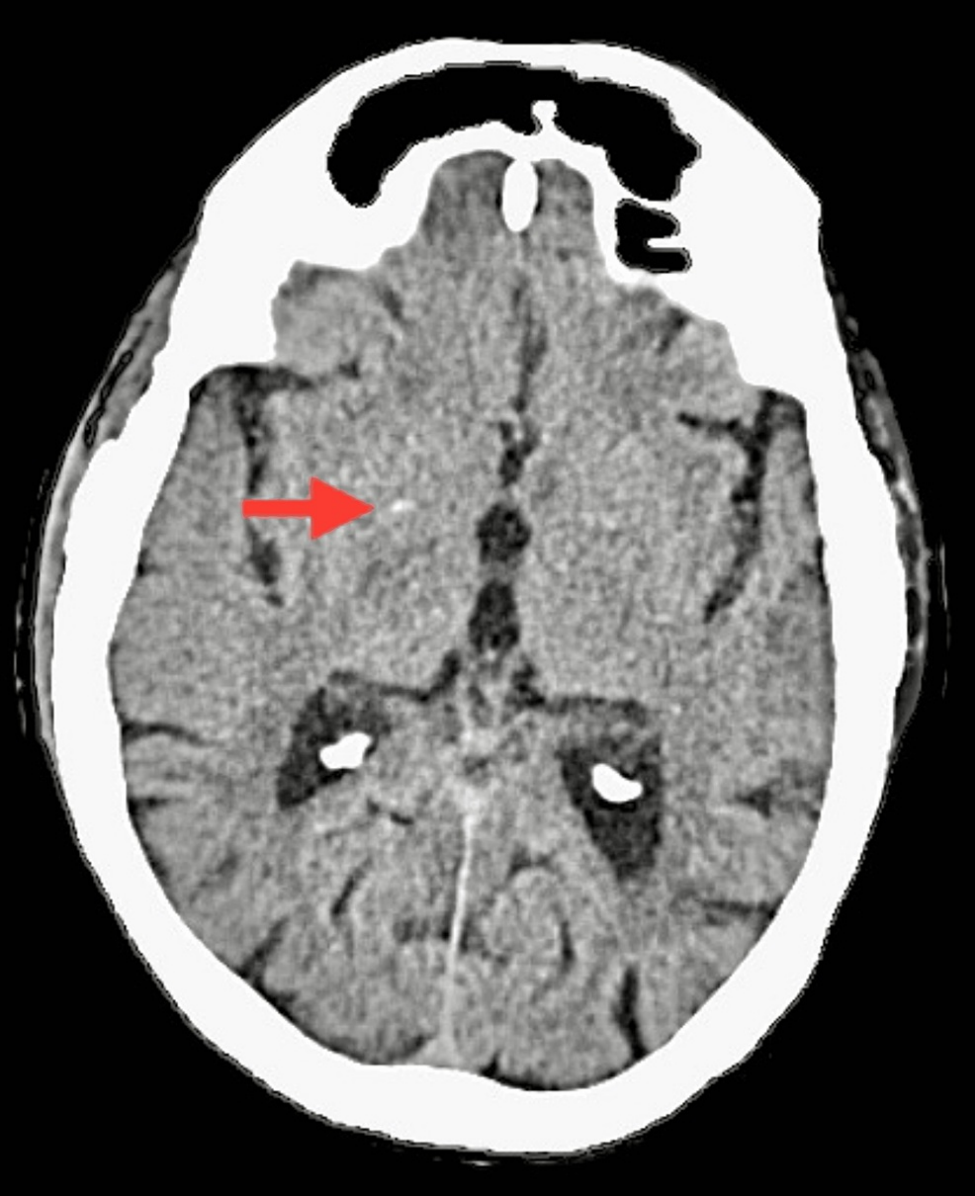
CT scan of the brain The red arrow shows an infarct in the right corona radiata.

**Figure 2:**
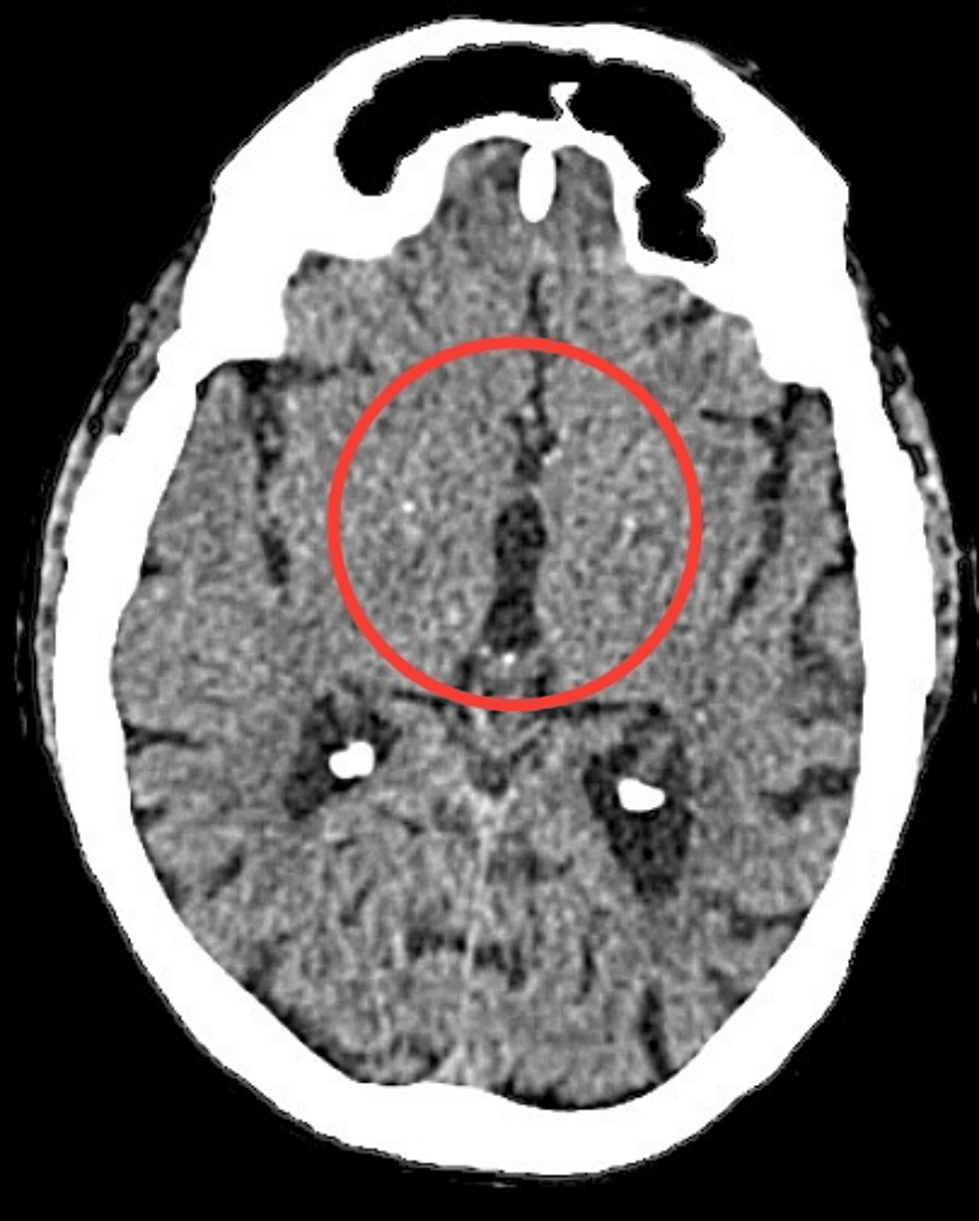
CT scan of the brain The red circle indicates chronic lacunar infarcts in the left gangliocapsular regions.

**Figure 3:**
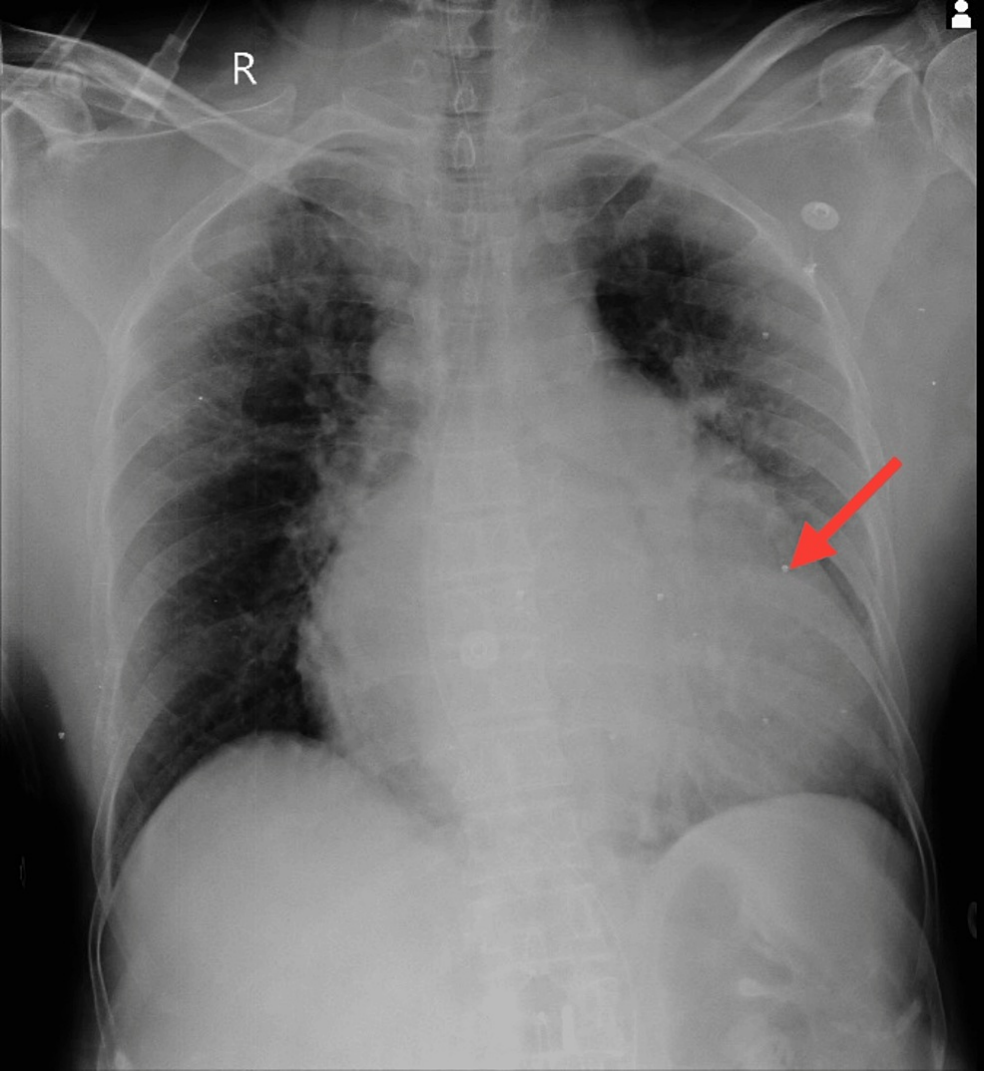
Chest X-ray eliciting cardiomegaly The red arrow shows cardiomegaly.

The patient was endomorphic and had normal vital signs. Notably, both the upper and lower limbs exhibited dry and scaly skin. Notable clinical findings suggested an altered mental state, as the patient was conscious but not oriented to her surroundings. Additionally, the presence of slurred speech raised concerns about potential neurological involvement. The Glasgow Coma Scale score was 13 (E4V4M5), indicating a moderate level of consciousness. Furthermore, the assessment of cardiac function revealed a low ejection fraction, emphasizing the need for a thorough investigation into the neurological and cardiovascular aspects of the patient’s condition. Color Doppler imaging indicated atherosclerotic changes in the bilateral common carotid and internal and external carotid arteries. MRI findings showed altered signal intensity in the right corona radiata, lentiform nucleus, and temporal region, along with age-related atrophy and small vessel ischemic disease on the left side. A CT scan of the brain revealed a lesion in the right corona radiata, as shown in Figure 1, and chronic lacunar infarcts in the left gangliocapsular regions, as shown in Figure 2. Chest X-ray supine revealed cardiomegaly, as shown in Figure 3. Neurological assessments showed exaggerated biceps jerk, absent knee jerk, and extensor plantar response on the left side, while reflexes were normal on the right. The tone was 2+ on the right side and 3+ on the left. The patient also exhibited Wernicke’s aphasia, adding to the complex clinical picture.

Therapeutic intervention

**Figure 4:**
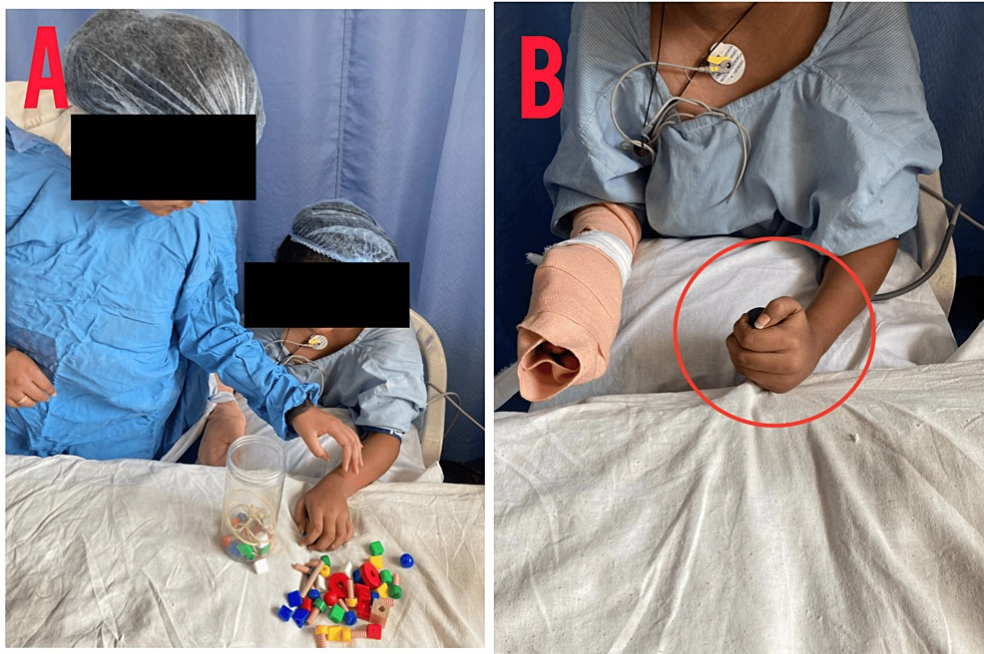
CIMT for the left upper limb (A) Bead exercises. (B) The red circle indicates squeezing a ball. CIMT, constraint-induced movement therapy

**Figure 5:**
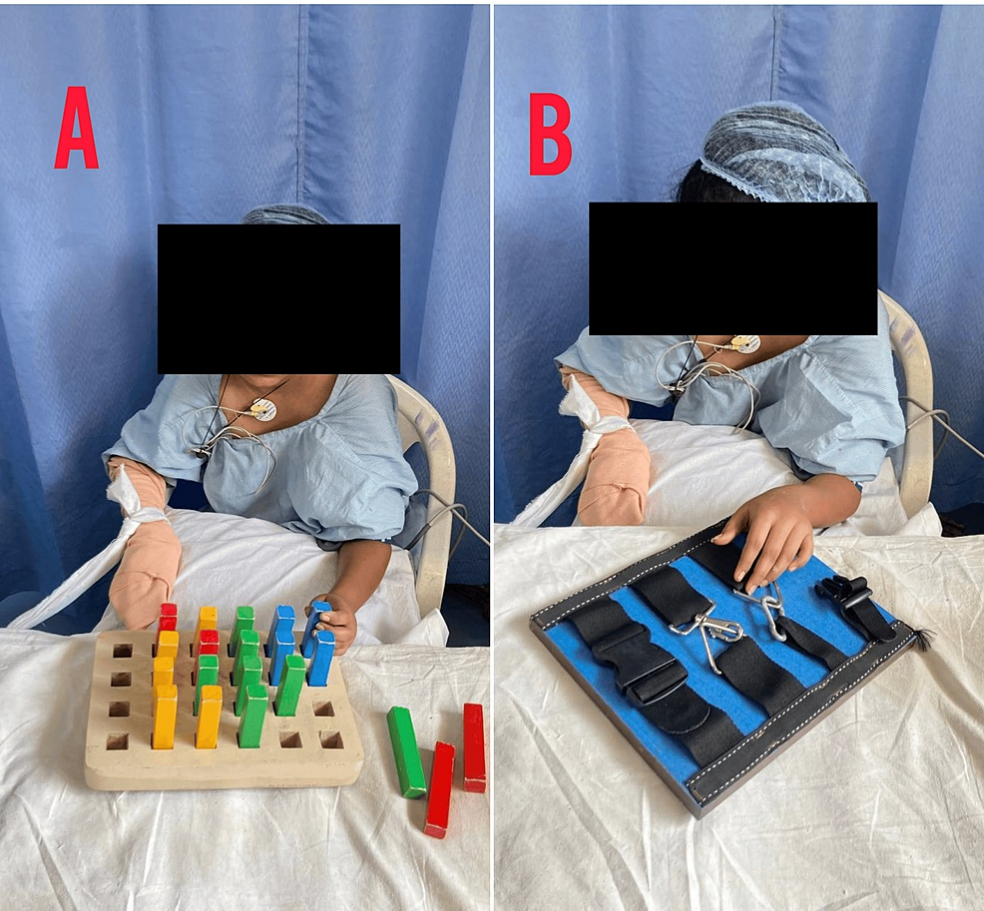
CIMT progressive exercises for the left upper limb (A) Pegboard exercises. (B) Opening and closing of zips in a zigzag pattern. CIMT, constraint-induced movement therapy

**Figure 6:**
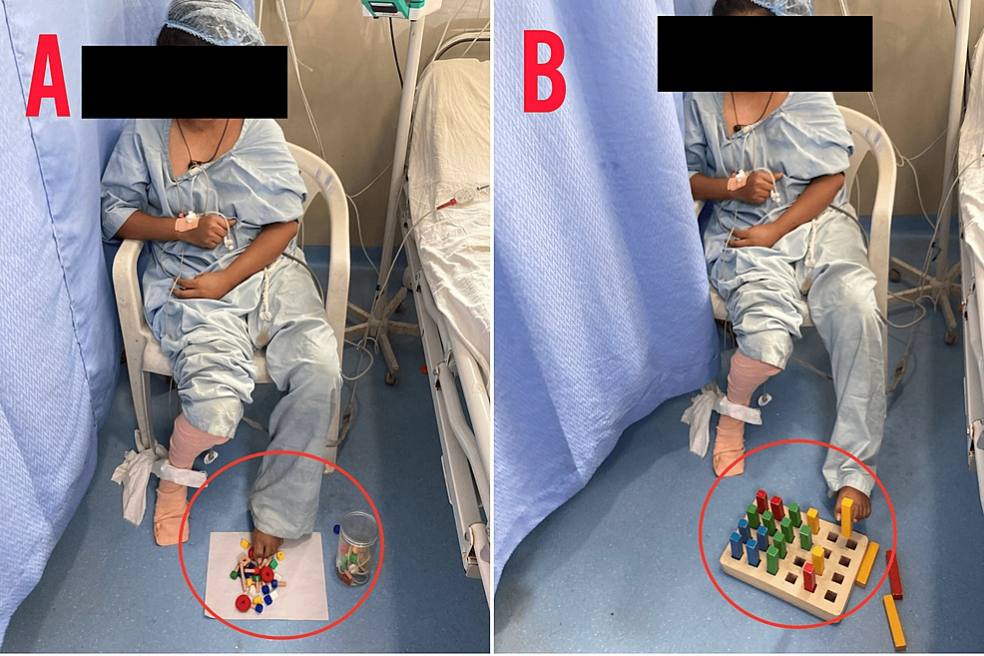
CIMT for the left lower limb (A) The red circle indicates bead exercises. (B) The red circle indicates pegboard exercises. CIMT, constraint-induced movement therapy

Along with conventional therapy, CIMT was planned for the patient, which encompasses restricting the non-paralyzed arm and using the paralyzed arm for task-oriented activities. Table 1 represents the conventional therapy given to the patient; Table 2 describes the CIMT given for the left upper limb; and Table 3 describes the CIMT given for the left lower limb. Figures 4, 5, 6 show the patient being rehabilitated.

**Table 1:** Conventional physiotherapy protocol utilized for recovery ADL, activities of daily living; AROM, active range of motion; PROM, passive range of motion

Phase	Goals	Interventions
Acute phase	Reduce edema and pain and prevent complications	Positioning to prevent contractures
		PROM exercises
		Bed mobility exercises
		Breathing exercises
		Limb elevation and effleurage
Subacute phase	Improve mobility and strength	AROM exercises
		Bed-to-chair transfer practice
		Gait training with assistance
		Strengthening exercises for the left side
Recovery phase	Enhance functional independence	Progressive resistance exercises
		Balance and coordination training
		ADL training with adaptive techniques
		Community reintegration activities

**Table 2:** CIMT for the left upper extremity CIMT, constraint-induced movement therapy

Week 1	Week 2	Week 3
Grasping beads	Practice putting beads into a box	Putting beads into a rope
Holding a squeeze ball for five minutes	Holding a bottle for five minutes	Holding a chair
Turning the pages of a notebook in a forward direction	Turning the pages of a newspaper in a forward direction	Turning the pages forward and backward alternatively
Transferring cotton balls from one bowl to another	Transferring cotton balls with the help of a tweezer	Transferring water directly from one glass to another
Grasping a needle	Making holes in a putty with a needle	Putting a needle into a piece of cloth
Opening and closing a drawer	Opening and closing a sliding door	Opening and closing a cupboard
Pegboard exercises	Continue pegboard exercises	Continue pegboard exercises
Putting a ball in a jar	Putting a key in a jar	Putting a button in a jar
Taking out clothes from a hanger	Hanging and taking out clothes from a hanger	Hanging, taking out, and wrapping clothes
Opening and closing zips in a horizontal or vertical direction	Opening and closing zips in a zigzag manner	Opening and closing zips in a straight and zigzag pattern, alternatively
Spreading butter on bread	Spreading cream on a cake	Painting a wall
Opening the cap of a jar	Opening the cap of a bottle	Sharpening a pencil

**Table 3:** CIMT for lower limbs CIMT, constraint-induced movement therapy

Week 1	Week 2	Week 3
Carrying beads with toes	Carrying a glass with toes	Pushing the wall
Spot marching with the affected leg	Walking with the affected leg and crossing obstacles	Walking backward with the affected leg and crossing obstacles
Walking straight	Walking in a zigzag pattern	Walking in a forward and zigzag pattern alternatively
Folding a handkerchief	Folding a T-shirt	Folding a saree
Putting a ball in a jar	Putting a key in a jar	Putting a button in a jar
Drawing a line with great toe	Drawing a semicircle	Drawing a circle
Pegboard exercises	Pegboard exercises	Pegboard exercises

Outcome measures

The outcomes of the intervention are shown in Table 4.

**Table 4:** Outcomes of interventions utilized for evaluation post-rehabilitation BBS, Berg Balance Scale; DGI, Dynamic Gait Index; FIM, Functional Independence Measure; STREAM, Stroke Rehabilitation Assessment of Movement

Outcome measures	Pre-rehabilitation	Post-rehabilitation
STREAM	17/70	30/70
DGI	Jul-24	Dec-24
FIM	131/210	180/210
BBS	May-56	21/56

## Discussion

In 1999, Miltner et al. conducted a clinical trial on CIMT in which the participants were 15 individuals. Nine people had a right-side stroke, and six had a left-side stroke; all were right-arm predominant prior to the stroke. The treatment consisted of two primary parameters using a resting hand splint for 90% duration. At the same time, the patient is awake for the whole span of 12 days for constriction of the movement of the non-impacted upper arm and training of the arm that was impacted through an action known as “shaping” for about seven hours per day on the eight weekdays during that time. Patients improved considerably from before to after therapy on a clinical motor examination and a test evaluating the extent of use of the diagnosed limb in day-to-day activities, with no regression in performance at the six-month follow-up [19]. In 2006, Wolf et al. conducted a randomized controlled trial in which respondents were allocated at random to CIMT, which involved using a hindering mitt on the less affected grasp while practicing and shaping behavior with the hemiplegic hand or conventional therapy, which included no post-rehabilitation treatment to pharmaceutical or physiotherapeutic interventions. Sex and pre-stroke dominance were used to stratify patients. Their findings showed that CIMT resulted in a statistically considerable and medically relevant increase in the quality of arm movements for people who had cerebrovascular accidents during the preceding three to nine months and had it for at least a year [17].

Choi et al. arranged a randomized controlled experiment in 2017 to evaluate if game-based CIMT improves equilibrium skills in people with cerebrovascular accidents. Thirty-six chronic stroke victims were randomly assigned to one of the following three categories: game-based CIMT (n = 12), general game-based training (n = 12), and traditional (n = 12). For four weeks, all techniques were carried out three times a week. The static balance control and weight-bearing symmetry were examined, as well as the Functional Reach Test (FRT), modified FRT, and Timed Up and Go Tests, to assess balancing abilities. According to the findings, while conventional game-based retraining and game-based CIMT enhanced static and dynamic equilibrium skills, game-based CIMT improved static equilibrium control, weight-bearing symmetry, and side-to-side weight shift to a greater extent [16]. Duarte Pereira et al. conducted a multiple case study in 2022, including a convenience sample of 12 participants (eight males) with an average age of 55 years old. The lower extremity CIMT (LE-CIMT) intervention process, combining task-oriented training, motor learning techniques, and a transfer package, was conducted in a clinical environment. CIMT increased gait metrics, reduced test execution time, and enhanced functional mobility in stroke patients, according to the findings. The study indicated that LE-CIMT is a valuable management strategy for increasing perceptions of improvements and encouraging paralyzed lower extremities in daily mobility tasks [20].

## Conclusions

According to studies, CIMT is the use of the paretic limbs by restricting non-paretic limbs and is mainly used for the upper limbs. Still, recent advances have proved that the therapy can be applicable for lower limbs to maintain balance and facilitate gait. Patients with stroke usually have difficulty performing activities of daily living due to weakness in their paretic limbs; hence, the therapy, which encompasses the use of paretic limbs, was found to be effective. Moreover, the subjective satisfactory experience with the CIMT provides valuable insights into the efficiency of this therapy in patients undergoing cerebrovascular accidents. The promising outcome measures have proved that CIMT in the ICU is a precise and versatile therapy for patients with stroke. Hence, we conclude that CIMT, in addition to primary rehabilitation, has been proven to be a promising approach for the rehabilitation of patients with stroke.

## References

[1] Johnson W, Onuma O, Owolabi M, Sachdev S (2016). Stroke: a global response is needed. Bull World Health Organ.

[2] Chemerinski E, Robinson RG (2000). The neuropsychiatry of stroke. Psychosoma.

[3] Albertson M, Sharma J (2014). Stroke: current concepts. S D Med.

[4] Bonita R, Mendis S, Truelsen T, Bogousslavsky J, Toole J, Yatsu F (2004). The global stroke initiative. Lancet Neurol.

[5] Béjot Y, Daubail B, Giroud M (2016). Epidemiology of stroke and transient ischemic attacks: current knowledge and perspectives. Rev Neurol (Paris).

[6] Feigin VL, Forouzanfar MH, Krishnamurthi R (2014). Global and regional burden of stroke during 1990-2010: findings from the Global Burden of Disease Study 2010. Lancet.

[7] Jang SH (2012). Motor recovery mechanisms in patients with middle cerebral artery infarct: a mini-review. Eur Neurol.

[8] Heiss WD (2016). Malignant MCA infarction: pathophysiology and imaging for early diagnosis and management decisions. Cerebrovasc Dis.

[9] Donzelli R, Marinkovic S, Brigante L, Divitiis O, Nikodijevic I, Schonauer C, Maiuri F (1999). Territories of the perforating (lenticulostriate) branches of the middle cerebral artery. Surg Radiol Anat.

[10] Arboix A, Bechich S, Oliveres M, García-Eroles L, Massons J, Targa C (2001). Ischemic stroke of unusual cause: clinical features, etiology and outcome. Eur J Neurol.

[11] Elkind MS, Sacco RL (1998). Stroke risk factors and stroke prevention. Semin Neurol.

[12] Murala S, Nagarajan E, Bollu PC (2022). Infectious causes of stroke. J Stroke Cerebrovasc Dis.

[13] Feske SK (2007). Stroke in pregnancy. Semin Neurol.

[14] Kwakkel G, Veerbeek JM, van Wegen EE, Wolf SL (2015). Constraint-induced movement therapy after stroke. Lancet Neurol.

[15] Taub E, Uswatte G, Pidikiti R (1999). Constraint-induced movement therapy: a new family of techniques with broad application to physical rehabilitation--a clinical review. J Rehabil Res Dev.

[16] Choi HS, Shin WS, Bang DH, Choi SJ (2017). Effects of game-based constraint-induced movement therapy on balance in patients with stroke: a single-blind randomized controlled trial. Am J Phys Med Rehabil.

[17] Wolf SL, Winstein CJ, Miller JP (2006). Effect of constraint-induced movement therapy on upper extremity function 3 to 9 months after stroke: the EXCITE randomized clinical trial. JAMA.

[18] Winstein CJ, Miller JP, Blanton S (2003). Methods for a multisite randomized trial to investigate the effect of constraint-induced movement therapy in improving upper extremity function among adults recovering from a cerebrovascular stroke. Neurorehabil Neural Repair.

[19] Miltner WH, Bauder H, Sommer M, Dettmers C, Taub E (1999). Effects of constraint-induced movement therapy on patients with chronic motor deficits after stroke: a replication. Stroke.

[20] Duarte Pereira N, Ilha J, Dos Anjos SM, Morris D (2022). Constraint-induced movement therapy for lower extremity use in activities of daily living in people with chronic hemiparesis: multiple case study. Int J Rehabil Res.

